# Gallic acid mitigates diclofenac-induced liver toxicity by modulating oxidative stress and suppressing *IL-1β* gene expression in male rats

**DOI:** 10.1080/13880209.2020.1777169

**Published:** 2020-07-07

**Authors:** Mohsen Esmaeilzadeh, Esfandiar Heidarian, Mehrnoosh Shaghaghi, Hoshang Roshanmehr, Mohammad Najafi, Alireza Moradi, Ali Nouri

**Affiliations:** a School of Biology, Damghan University, Damghan, Iran; b Clinical Biochemistry Research Center, Basic Health Sciences Institute, Shahrekord University of Medical Sciences, Shahrekord, Iran; c Department of Biology, Faculty of Basic Science, Tehran Payamenoor University, Tehran, Iran; d Student Research Committee, Ahvaz Jundishapur University of Medical Sciences, Ahvaz, Iran; e Department of Biochemistry, Faculty of Medicine, Iran University of Medical Sciences, Tehran, Iran; f Department of Physiology, Iran University of Medical Sciences, Tehran, Iran

**Keywords:** Free radical, Protein carbonyl, GPx, Catalase, MDA

## Abstract

**Context:**

Diclofenac (DIC) is an NSAID and consumption of this drug creates side effects such as liver injury. Gallic acid (GA), a natural component of many plants, is used as an antioxidant agent.

**Objective:**

This study assesses the hepatoprotective effects of GA in the rat model of DIC-induced liver toxicity.

**Materials and methods:**

In this research, the male Wistar rats were separated into five groups (*n* = 6). Group 1, control, received normal saline (1 mL/kg bw, i.p.); Group 2 received DIC-only (50 mg/kg bw, i.p.); Groups 3, received DIC (50 mg/kg bw, i.p.) plus silymarin (100 mg/kg bw, po), groups 4 and 5 received DIC (50 mg/kg bw, i.p.) plus GA (50 and 100 mg/kg, po, respectively).

**Results:**

The data demonstrated that the liver levels of the GSH, GPx, SOD, and CAT significantly reduced and the levels of the serum protein carbonyl, AST, ALP, ALT, total bilirubin, MDA, serum IL-1β, and the liver *IL-1β* gene expression were remarkably increased in the second group compared to control group. On the other hand, treatment with GA led to a significant elevation in GSH, GPx, SOD, CAT, and a significant decrease in protein carbonyl, AST, ALP, ALT, total bilirubin, MDA, serum IL-1β, and gene expression of *IL-1β* in comparison with the second group. Histological changes were also ameliorated by GA oral administration.

**Discussion and Conclusions:** The data show that the oral administration of GA could alleviate the noxious effects of DIC on the antioxidant defense system and liver tissue.

## Introduction

The liver is a vital organ that has various roles in the body, including the production of proteins, manufacturing triglycerides and cholesterol, glycogen synthesis, and bile creation. The liver is involved in metabolising various toxins, including chemicals, drugs, and natural substances (Jameson 2018). Nonsteroidal anti-inflammatory drugs (NSAIDs) are commonly applied for relieving inflammatory illnesses and pain. Therapeutic doses of NSAIDs generally produce only mild side effects, however, overdoses of these drugs can produce severe toxicity. The majority of NSAIDs inhibit COX enzymes and consequently change prostaglandin production, and as a result, liver and kidney cells are exposed to injury (Alabi and Akomolafe 2020; Besen et al. 2009; Chandrasekharan et al. 2002). Diclofenac (DIC) is an NSAID with analgesic, anti-inflammatory, antipyretic, and antinociceptive attributes (Mazumdar et al. 2006). It is widely used in the treatment of rheumatoid arthritis pain. In spite of the therapeutic profits of DIC, it has serious severe side effects, including gastrointestinal injury and damage to hepatic, renal, lungs, and cardiac tissues (Harirforoosh et al. 2016; Tomic et al. 2008). Production of reactive oxygen species (ROS) and reactive metabolites has been considered as the major cause of DIC-induced hepatotoxicity. Cytochrome P450-dependent oxidation of DIC creates 2,5-quinone imines and 4′,5-hydroxy-diclofenac (Al-dossari et al. 2020). These factors can cause covalent alteration of proteins and conjugate with glutathione (GSH) to produce adducts of GSH (Tang 2003). The metabolites of DIC can elevate the production of ROS via the induction of redox imbalance and mitochondrial dysfunction (Moreno-Sánchez et al. 1999). Hepatocyte damage by DIC’s generation of extra ROS has been well documented (Elgebaly et al. 2018; Mahmoud et al. 2017). Thus, it is reasonable to assume that high levels of antioxidants would be required to ameliorate DIC-induced toxicity.

Gallic acid (GA) is a naturally occurring compound of many plants that is used in the food, cosmetics, and drug industries. GA is widely found throughout the plant kingdom and is a well-known antioxidant (Kim 2007; Priscilla and Prince 2009), furthermore, there exists a large volume of scientific evidence documenting its antibacterial, antiviral, antifungal, anticancer, and antioxidant activities (Nabavi et al. 2013; Omobowale et al. 2018). Nevertheless, based on our review of the literature, the effects of GA on DIC-induced liver toxicity and oxidative stress have not been investigated. In this study, we evaluated the ameliorative effect of GA against DIC-induced liver toxicity and oxidative stress.

## Materials and methods

### Chemicals

2-Thiobarbituric acid (TBA), sodium acetate, and H_2_O_2_ were procured from Merck (Darmstadt, Germany). Greiss reagent, gallic acid, 5,5-dithiobis-2-nitrobenzoic acid, nitro blue tetrazolium, and TPTZ (2,4,6-tripyridyl-s-triazine), and 2,4-dinitrophenylhydrazine were obtained from Sigma-Aldrich Company (St. Louis, MO, USA). SYBRR Green Real Time-PCR Master Mix was purchased from the Qiagen Co. (Dusseldorf, Germany). The IL-1β kit used was procured from the Bioassay Technology Laboratories (BT Lab) in China. Total bilirubin, ALP (alkaline phosphatase), ALT (alanine aminotransferase) and AST (aspartate aminotransferase) kits were provided by Pars Azmoon Co. (Tehran, Iran).

### Animals

Thirty male Wistar rats weighting 200 ± 20 g (6–8-week old) obtained from the Tehran Pasteur Institute (Tehran, Iran) were used. The rats were kept in standard laboratory conditions, including 25 °C ambient temperature, 50% relative humidity, and a 12 h dark/light cycle, and were allowed free access to water and standard diet. The whole protocol of the research was approved by Shahrekord University of Medical Sciences Ethics Committee, Shahrekord, Iran (Ethic number, IR. SKUMS. REC. 1394. 146).

### Study design

The thirty male Wistar rats were randomly placed into 5 groups (*n* = 6). Group 1 was the control group receiving normal saline (1 mL/kg body weight (bw), i.p.) and 1 mL distilled water as the solvent of GA by gavage at an interval of 1 h for 5 days to provide an equal shock in the control group as opposed to other groups. Group 2 received DIC only (50 mg/kg bw, i.p.) and 1 mL distilled water (solvent of GA, po) at an interval of 1 h for 5 days (Aydin et al. 2003; Peter et al. 2017). Group 3 received DIC (50 mg/kg bw, i.p.) and 1 mL of silymarin (100 mg/kg bw, po) at an interval of 1 h for 5 days (Peter et al. 2017; Heidarian and Nouri 2019). Groups 4 and 5 received DIC (50 mg/kg bw, i.p.) and 1 mL of GA (50 and 100 mg/kg, po, respectively) at an interval of 1 h for 5 days (Jadon et al. 2007; Padma et al. 2011). At the end of the study, rats were fasted for 12 h and then sacrificed under mild anaesthesia. Whole blood specimens were collected by cardiac puncture method to gain plasma and serum. In addition, liver tissues were separated for obtaining liver SOD, CAT activities, lipid peroxidation (LPO), IL-1β gene expression, and histological studies.

### Determination of serum biochemical parameters

Total bilirubin, ALP, ALT, and AST liver enzymes were determined using an auto-analyzer system (BT3000, Rome, Italy).

### Determination of plasma antioxidant capacity

Plasma antioxidant capacity calculated by ferric reducing/antioxidant power (FRAP) method as described previously (Nouri et al. 2019).

### Determination of nitrite content

Determining of nitrite content was done by using Greiss reagent as described previously (Heidarian and Nouri 2019).

### Determination of lipid peroxidation (LPO) and serum protein carbonyl (PC)

Serum and liver MDA levels were determined by the TBA method as previously described by Heidarian and Soofiniya (2011). The serum PC was assessed using Reznick and Packer (1994) protocol at 360 nm with 6 M guanidine hydrochloride. The results were demonstrated in nmol dinitrophenyl hydrazine (DNPH)/mg protein.

### Determination of tissue CAT and SOD activities

The liver CAT activity of experimental groups was measured as described previously (Heidarian et al. 2014). The activity of liver SOD was evaluated using the inhibition of the nitro blue tetrazolium (NBT) photochemical reaction at 560 nm (Flohe 1984). All total protein samples were assessed using the method of Bradford (1976). Data were described as U/mg protein.

### Determination of tissue GSH levels

Reduced GSH content was assayed according to the Ellman (1959) method. The GSH reacts with 5,5-dithiobis-2-nitrobenzoic acid and the absorbance spectra have a peak value at 410 nm. The data were demonstrated as μmol/g tissue.

### Tissue GPx activity

GPx activity was evaluated by determining the reduction in GSH content after incubating the sample in the presence of H_2_O_2_ and NaN_3_ (Hafeman et al. 1974).

### Determination of serum IL-1β

The serum level of IL-1β was assessed using the ELISA assay kit according to the manufacturer’s instructions. Data were reported as pg/mL.

### Real-time RT-PCR analysis

The liver mRNA was extracted using the BIOZOL kit reagent (Bioer, China) according to the instructions. The quality and quantity of total RNA were assessed by reading absorbance at 260/280 nm using a spectrophotometer (Nanodrop2000, Thermo, USA). cDNA measurement was accomplished using a PrimeScript^TM^ reagent kit (Takara Bio Inc. Japan**)** in accordance with the manufacturer’s instructions. Then, cDNA was amplified according to RT-qPCR using SYBR® Green PCR Master Mix in the presence of IL-1β primer (forward sequence 5′-CAACAAAAATGCCTCGTGCTG-3′ and reverse sequence 5′-TCGTTGCTTGTCTCTCCTTGTA-3′) and β-actin primer (forward sequence 5′-CGCAAATTACCCACTCCCGAC-3′ and reverse sequence 5′-GTAACCTCCCGTTCAGACCAC-3′). The primers were designed by Oligo 7.0 software and they were confirmed by Blast Nucleotide (NCBI). PCR carried out in primary denaturation at 95 °C for 10 min. RT-q PCR was performed in 40 cycles (including secondary denaturation at 95 °C for 15 sec, annealing at 60 °C for 20 sec, and extension at 72 °C for 25 sec). β-Actin gene was used as an internal control gene to control the expression of mRNA. Further, the ΔΔCT method was used for the analysis of gene expression.

### Histopathological study

After sacrificing the rats, their livers were fixed in 10% formaldehyde solution and were dissected by a microtome (AMR 400, Amos Scientific, Australia) in 5 μm slices of tissue, and after paraffin embedding, stained with haematoxylin, and eosin (H&E) (Carleton et al. 1980). The liver histopathological examinations, routine haematoxylin and eosin (H&E) staining, were performed. An optical microscope (Nikon Eclipse E400 microscope with a digital camera, USA) was used to evaluate tissue changes.

### Statistical analysis

One-way analysis of variance (ANOVA) was used for data analysis using SPSS software (Statistical Package for the Social Sciences, version 20.0, SPSS Inc, Chicago, IL). All data were expressed as mean ± SD. The mean values of the groups were compared using the Tukey’s *post hoc* test. *p* < 0.05 was considered a significance level.

## Results

### Effect of gallic acid on serum ALT, AST, ALP and total bilirubin

The results indicated that injection of DIC for 5 days led to a significant increase (*p* < 0.05) in total bilirubin, AST, ALP, and ALT in rats received DIC-alone compared to the control group (Table 1). Treatment with GA at doses of 50 and 100 mg/kg reduced the serum levels of above mentioned parameters when compared with the group received DIC-alone. Total bilirubin, AST, ALP, and ALT levels decreased significantly in the rats received 100 mg/kg GA (*p* < 0.05), compared to those received 50 mg/kg GA.

**Table 1.** Effect of gallic acid on serum alanine aminotransferase (ALT), aspartate aminotransferase (AST), alkaline phosphatase (ALP) and total bilirubin.

Parameters	Group 1	Group 2	Group 3	Group 4	Group 5
**ALT** (U/L)	65.2 ± 5.1	136.8 ± 11.7^a^	66.2 ± 9.8^b^	96.1 ± 8.1^a^^bc^	68.9 ± 11.1^b^^d^
**AST** (U/L)	138.5 ± 16.7	286.7 ± 17.5^a^	144.6 ± 15.7^b^	198.7 ± 14.6^a^^bc^	143.4 ± 13.7^b^^d^
**ALP** (U/L)	180.2 ± 19.5	458.6 ± 45.1^a^	189.9 ± 22.1^b^	281.3 ± 34.2^a^^bc^	186.3 ± 21.2^b^^d^
**Total bilirubin** (mg/dl)	0.84 ± 0.08	2.32 ± 0.42^a^	0.82 ± 0.12^b^	1.42 ± 0.25^a^^bc^	0.91 ± 0.13^b^^d^

Data are expressed as mean ± SD (*n* = 6) and analysed by one-way ANOVA followed by Tukey post hoc test. Group 1: control group; Group 2: diclofenac-alone treated group; Group 3: treated by diclofenac plus silymarin (100 mg/kg p.o.); Groups 4 and 5 were treated by diclofenac plus gallic acid (50, 100 mg/kg p.o. respectively).

^a^*p* < 0.05 versus control group (Group 1),

^b^*p* < 0.05 versus diclofenac-alone treated group (Group 2),

^c^*p* < 0.05 versus group treated with silymarin (Group 3),

^d^*p* < 0.05 versus group treated with gallic acid at dose of 50 mg/kg (Group 4).

### Effect of gallic acid on plasma antioxidant capacity, nitrite content and MDA levels

Table 2 shows that injection of DIC in the DIC-alone treated group caused a remarkable decrease (*p* < 0.05) in plasma antioxidant capacity and a noticeable increased in nitrite level, liver MDA, and serum MDA contents compared to the control animals (group 1). On the other hand, treatment with GA at doses of 50 and 100 mg/kg caused a noticeable raised (*p* < 0.05) in plasma antioxidant capacity and a noticeable decreased (*p* < 0.05) in nitrite content, serum MDA and liver MDA levels compared to the DIC-alone treated group. A significant change (*p* < 0.05) in plasma antioxidant capacity, nitrite content, serum MDA and liver MDA between rats received GA in doses of 50 and 100 mg/kg was found.

**Table 2.** Effect of gallic acid on ferric reducing/antioxidant power (FRAP), malondialdehyde (MDA), and protein carbonyl (PC) levels.

Parameters	Group 1	Group2	Group 3	Group 4	Group 5
Plasma FRAP (μM)	661.2 ± 60.1	332.1 ± 33.4^a^	671.2 ± 51.6^b^	518.1 ± 41.2^a^^bc^	658.8 ± 49.6^b^^d^
Serum MDA (nmol/L)	9.12 ± 0.62	20.14 ± 2.11^a^	9.42 ± 1.06^b^	14.61 ± 1.69^a^^bc^	9.58 ± 1.32^b^^d^
Liver MDA (nmol/mg protein)	1.38 ± 0.21	5.46 ± 1.05^a^	1.42 ± 0.29^b^	2.98 ± 0.63^a^^bc^	1.51 ± 0.38^b^^d^
Serum PC (nmolNADPH/mg protein)	5.11 ± 0.71	12.61 ± 1.12^a^	4.98 ± 0.89^b^	8.28 ± 1.29^a^^bc^	5.23 ± 0.91^b^^d^

Data are expressed as mean ± SD (*n* = 6) and analysed by one-way ANOVA followed by Tukey post hoc test. Group 1: control group; Group 2: diclofenac-alone treated group; Group 3: treated by diclofenac plus silymarin (100 mg/kg p.o.); Groups 4 and 5 were treated by diclofenac plus gallic acid (50, 100 mg/kg p.o. respectively).

^a^*p* < 0.05 versus control group (Group 1),

^b^*p* < 0.05 versus diclofenac-alone treated group (Group 2),

^c^*p* < 0.05 versus group treated with silymarin (Group 3),

^d^*p* < 0.05 versus group treated with gallic acid at dose of 50 mg/kg (Group 4).

### Effect of gallic acid on serum protein carbonyl (PC)

Serum PC content increased considerably (*p* < 0.05) in the rats injected with DIC, compared to the control group (Table 2). However, the serum PC level in rats administrated with two different doses of GA (50 and 100 mg/kg) and a single dose of silymarin (100 mg/kg) significantly decreased (*p* < 0.05) compared to the rats injected with DIC-only. The serum PC level was remarkable (*p* < 0.05) between the fourth and fifth groups (rats administered with 50 and 100 mg/kg bw GA, respectively).

### Effect of gallic acid on CAT, SOD, GPx activities and GSH level

Table 3 shows that the injection of DIC led to a remarkably decrease (*p* < 0.05) in hepatic CAT and SOD activities in the DIC-alone treated group relative to the control animals. However, treatment with GA at doses of 50 and 100 mg/kg remarkably elevated liver SOD and CAT activities relative to the DIC-alone treated group. Table 3 also indicated a noticeable (*p* < 0.05) decline in liver GPx activity in the DIC-alone treated group compared to the control animals. However, the administration of GA at doses of 50 and 100 mg/kg caused a remarkable increase (*p* < 0.05) in liver GPx activity in comparison with the DIC-alone injected group. Also, injection of DIC in the DIC-alone injected group caused a noticeable decline (*p* < 0.05) in liver GSH compared to the control group (Table3). However, in groups treated with doses of 50 and 100 mg/kg GA (groups 4 and 5), liver GSH noticeably elevated (*p* < 0.05) compared to the second group (DIC-alone injected group).

**Table 3.** Effect of gallic acid on catalase (CAT) activity, superoxide dismutase (SOD) activity, glutathione peroxidase (GPx) activity and Intracellular glutathione (GSH) level.

Parameters	Group 1	Group2	Group 3	Group 4	Group 5
CAT (U/mg protein)	176 ± 14.9	51.7 ± 8.4^a^	168.2 ± 27.6^b^	113.4 ± 19.7^a^^bc^	179.2 ± 16.2^b^^d^
SOD (U/mg protein)	34.1 ± 2.9	15.2 ± 1.3^a^	32.6 ± 2.6^b^	21.6 ± 2.1^a^^bc^	31.3 ± 1.8^b^^d^
GPx (U/mg protein)	27.1 ± 1.0	19.3 ± 0.7^a^	26.8 ± 0.8^b^	23.4 ± 0.9^a^^bc^	27.8 ± 1.1^b^^d^
GSH (μmol/g tissue)	13.6 ± 0.2	5.2 ± 0.1^a^	13.8 ± 0.4^b^	9.8 ± 0.1^a^^bc^	13.2 ± 0.3^b^^d^

Data are expressed as mean ± SD (*n* = 6) and analysed by one-way ANOVA followed by Tukey post hoc test. Group 1: control group; Group 2: diclofenac-alone treated group; Group 3: treated by diclofenac plus silymarin (100 mg/kg p.o.); Groups 4 and 5 were treated by diclofenac plus gallic acid (50, 100 mg/kg p.o. respectively).

^a^*p* < 0.05 versus control group (Group 1),

^b^*p* < 0.05 versus diclofenac-alone treated group (Group 2),

^c^*p* < 0.05 versus group treated with silymarin (Group 3),

^d^*p* < 0.05 versus group treated with gallic acid at dose of 50 mg/kg (Group 4).

### Effect of gallic acid on serum level and gene expression of IL-1β

**Figure 1.**
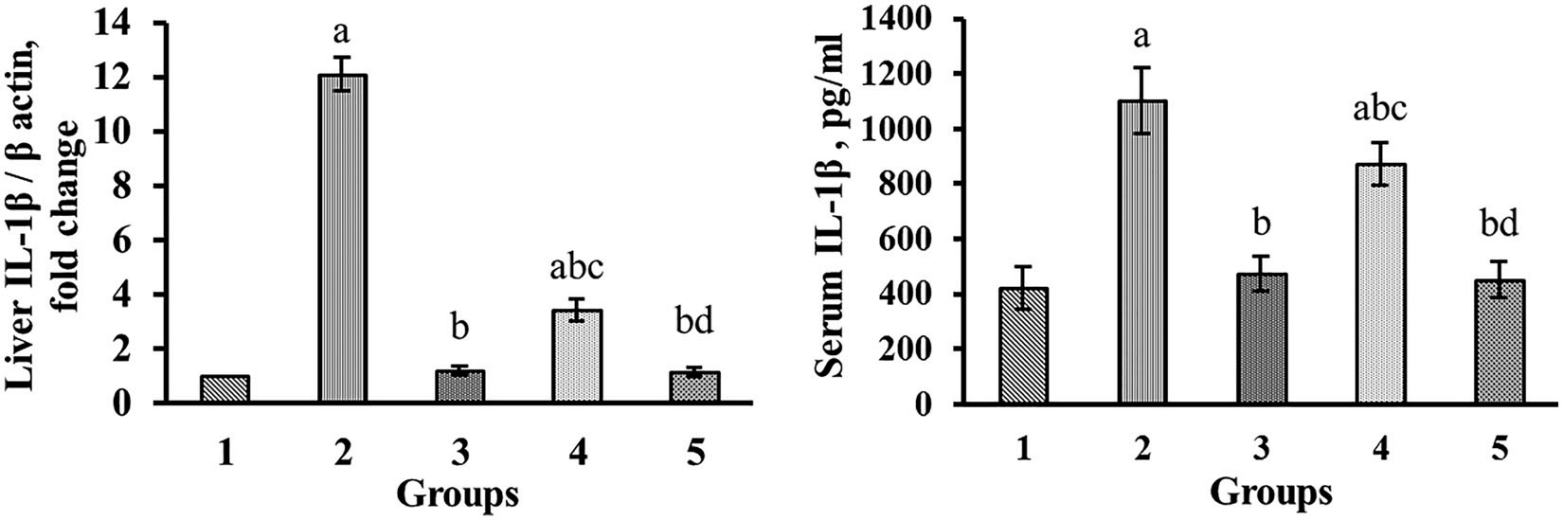
Effect of gallic acid on serum interleukin 1 beta (IL-1β) and expression of *IL-1β.* Data are expressed as mean ± SD (*n* = 6). Group 1: control group; Group 2: diclofenac-alone treated group; Group 3: treated by diclofenac plus silymarin (50 mg/kg p.o.); Groups 4 and 5 were treated by diclofenac plus gallic acid (50, 100 mg/kg p.o. respectively). ^a^*p* < 0.05 versus control group (Group 1), ^b^*p* < 0.05 versus diclofenac-alone treated group (Group 2), ^c^*p* < 0.05 versus group treated with silymarin (Group 3 ), ^d^*p* < 0.05 versus group treated with gallic acid at dose of 50 mg/kg (Group 4).

Figure 1 indicated IL-1β fold change expression in the studied groups. The results of this study indicated that the expression of the IL-1β gene in the DIC-only treated group increased in comparison with the control group (*p* < 0.05). However, in the treated groups with GA in doses of 50, and 100 mg/kg the expression of this gene reduced noticeably (*p* < 0.05) compared to the DIC-alone treated group. GA was able to reduce the IL-1β level at a dose of 100 mg/kg more than the other dose. Also, the injection of DIC in the DIC-alone treated group resulted in a significant increase (*p* < 0.05) in the serum level of IL-1β relative to the control group (Figure 1). However, in groups treated with GA at doses of 50, and 100 mg/kg the serum level of this protein significantly (*p* < 0.05) decreased compared to the DIC-alone treated group. There was no significant difference between the group treated with 100 mg/kg GA and control group.

### Histopathological findings

**Figure 2.**
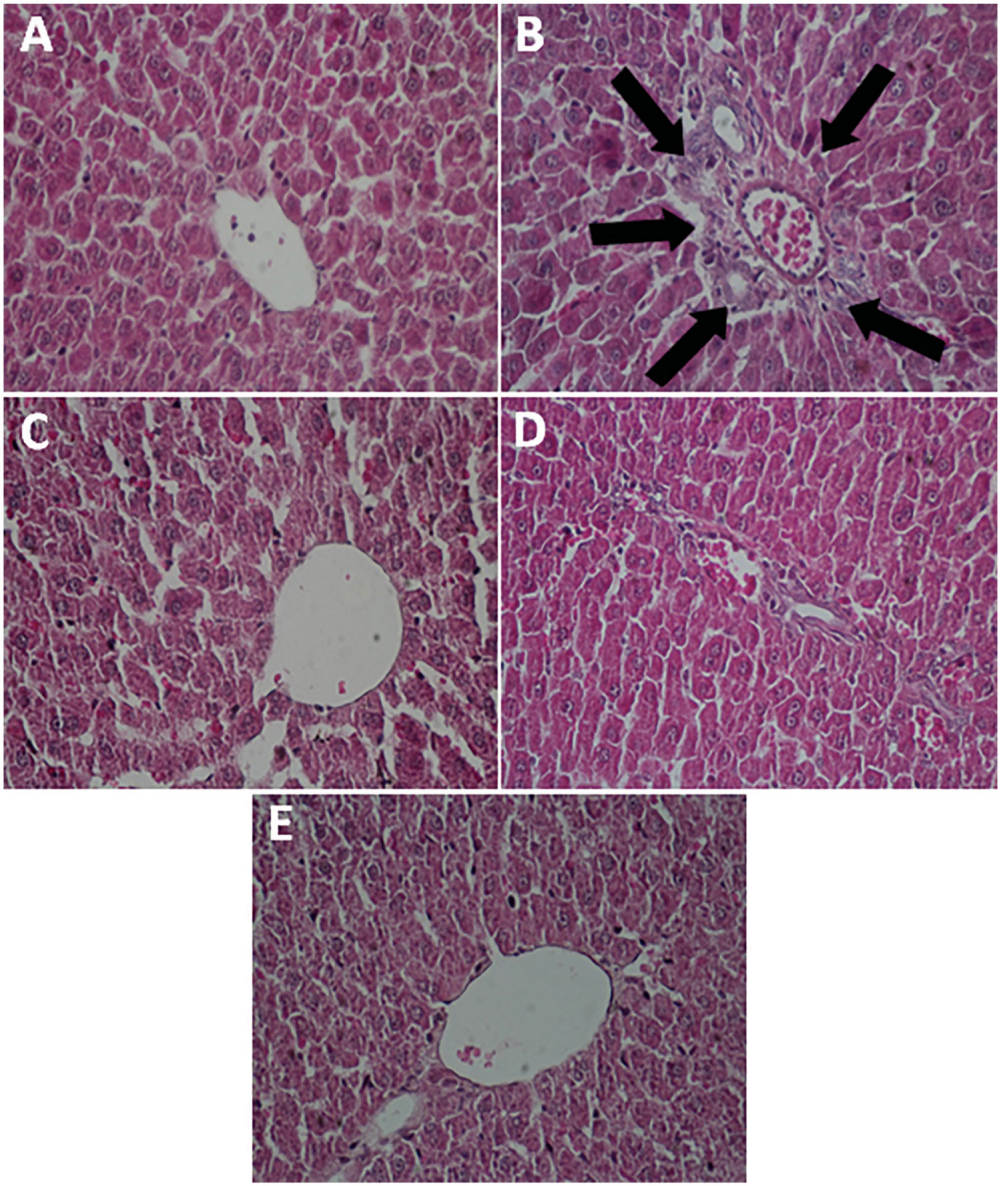
Effects of gallic acid on the liver histology of experimental groups. (A) Control group with normal structure; (B) diclofenac-alone treated rats; (C) diclofenac-injected rats supplemented with silymarin (100 mg/kg bw); (D) diclofenac-injected rats supplemented with gallic acid (50 mg/kg bw); (E) diclofenac-injected rats supplemented with gallic acid (100 mg/kg bw). The black arrows show lymphocyte infiltration.

Figure 2 indicated the representative histopathological studies in the experimental groups. Histology showed the normal morphology of the hepatocytes in the control group (Figure 2(A)). Injection of DIC in the DIC-alone treated group led to the infiltration of lymphocyte cells in comparison with the control group (Figure 2(B)). Also, DIC‐injected rats supplemented with silymarin showed decreased inflammatory cell infiltration relative to the DIC-alone treated group (Figure 2(C)). Liver degeneration and lymphocytic cell infiltration were significantly reduced in groups treated with GA at doses of 50 and 100 mg/kg compared to the DIC-alone treated group (Figure 2(D,E)).

## Discussion

Some drug metabolism and detoxification are accompanied by GSH conjugation that results in decrease antioxidant status and accumulation of toxic metabolites in the liver. The liver toxicity effects of diclofenac (DIC) in both animal models and humans have been described (Björnsson 2016; Licata 2016). Therefore, discovery effective method for diminishing liver fibrosis and inhibiting the progression of cirrhosis is very important.

One of the key markers of liver damage is the release of some enzymes such as ALP, ALT, and AST into the circulatory blood system. In this study significant increase observed in the levels of ALT, AST, ALP, and total bilirubin content in DIC-only treated animals in comparison with control animals confirmed the hepatotoxic potential of DIC (Table 1). This is in accordance with prior studies showing an elevation in ALT, AST, ALP, and total bilirubin levels in DIC-exposed humans and experimental animals (Adeyemi and Olayaki 2018; Alabi et al. 2017; Nouri et al. 2019). The diminution levels of total bilirubin, AST, ALT, and ALP in gallic acid (GA) and silymarin treated groups suggested that both components have protective effects against DIC-induced hepatotoxicity. These findings are sustained by the prior findings, which have indicated that GA attenuates selected drug and chemical-induced hepatotoxicity (Karimi-Khouzani et al. 2017; Latief et al. 2016).

Malondialdehyde (MDA) level is a sign of lipid peroxidation (LPO) (Gaweł et al. 2004). In the present study, the levels of MDA were significantly increased in the DIC-alone administrated rats compared to the control group (Table 2), which is in agreement with prior investigations (Alabi et al. 2017; Ramezannezhad et al. 2019). In addition, the administration of GA not only led to an increase in FRAP content in treated groups compared to the DIC-alone treated group, but also let to a decrease in MDA level in the serum and hepatic tissues (Table 2). The raised FRAP and declined LPO level in GA treated groups might be due to its free radical scavenging property.

CAT and SOD are vital enzymes of the antioxidant defense system. The SOD fuse two superoxide radicals (O_2_^•−^) and creates H_2_O_2_. H_2_O_2_ is eventually transformed into H_2_O and oxygen molecule by CAT in the peroxisomes (Sharifinasab et al. 2016; Wei et al. 2014). Various investigations have indicated that DIC could decline the actions of antioxidant enzymes in the liver (Ahmad et al. 2013; Nouri et al. 2019). GA as an antioxidant agent could noticeably raise the activity of antioxidant enzymes (Table 3). It confirms the prior findings that disclosed the anti-oxidative potential of GA (Karimi-Khouzani et al. 2017; Latief et al. 2016). The decline of SOD and CAT activities caused by DIC injection was manifest by elevated oxidative stress. However, GA treatment eventually reduced the MDA and elevated the activities of antioxidant defense system enzymes in hepatic tissue, reflecting its antioxidant potential mediated by hepatoprotective activity.

In addition, the excessive production of ROS following DIC exposure was related to the high levels of protein oxidation reaction, leading to the formation of protein carbonyl (PC) levels, signifying that oxidative protein damage might be one of the mechanisms of DIC induced liver toxicity. These observations are in line with previous investigations (Nouri and Heidarian 2019; Nouri et al. 2017). In the present study, the treatment with GA prevented oxidative damage induced by DIC in the liver tissue was along with a decrease in the level of PC (Table 3). In fact, GA acts as a free radical scavenger and is able to stabilise membrane structures.

Glutathione (GSH), an endogenous antioxidant, plays a main role in cellular defense in damage caused by oxidative stress. Both *in vitro* and *in vivo* reports have implicated the role of GSH depletion in oxidative stress induced by DIC in different model systems (Ahmad et al. 2013; Alabi et al. 2017; Niu et al. 2015). In the present study, DIC-induced liver toxicity caused a remarkable elevation in the GSH content in liver tissues (Table 3) compared to the control group. On the other hand, a noticeable restoration of GSH level observed in GA treated groups. The restoration of GSH level induced by GA might be either due to a direct increase in GSH content or due to decreased oxidative stress.

In order to evaluate the effect of GA on GSH metabolism, this study assessed its effect on GPx as GSH metabolising enzyme. GPx simplifies the neutralisation of peroxides through conjugation with GSH and leading to raised levels of oxidised glutathione (GSSG), which is then reduced by GR back to the sulfhydryl form (GSH) thus sustaining the antioxidant level. DIC-induced variations in GPx activity have been described prior to liver and kidney tissues (Ahmad et al. 2013; Peter et al. 2017). In the present study, DIC-induced reduction of GPx activity (Table 3) may be due to the defense mechanism against diminution lipid peroxidation and a noticeable rise in GPx activity in the administrated groups with GA can be due to the reduction content of LPO or oxidative stress.

Some investigations have also shown that DIC can induce circulation of macrophages and monocytes, which leads to the synthesis and release of a variety of pro-inflammatory cytokines including TNF-α and IL-1β (Peter et al. 2017; Nouri et al. 2019). Many findings also revealed that IL-1β plays a vital role in the maintenance and development of inflammation, and cytokines elevation is associated with liver injury (Abd-Ellah 2016; Sultan et al. 2017). The results of this research demonstrated that DIC administration markedly upregulated the *IL-1β* expression in the liver tissue (Figure 1). However, GA markedly decreased *IL-1β* up-regulation. The results of this work noticed that GA could ameliorate the liver injury caused by DIC via suppressing the inflammatory response. When tissue damage happens, leucocytes quickly migrate to sites of damage and initiate an inflammatory response. Accordingly, the infiltration of leucocyte was considered a sign of inflammatory response (Laskin and Laskin 2001; Pang et al. 2001). As already revealed in histopathological findings, leukocyte infiltration was significantly elevated in the liver of DIC-exposed rats. However, in our study treatment with GA efficaciously reduced leucocyte infiltration in the liver of the rats receiving DIC (Figure 2). Therefore, histopathological findings indicated that GA could decrease the DIC-induced inflammatory response in the liver tissue.

## Conclusions

The present study indicated that GA has ameliorative effects against DIC-induced liver toxicity by reducing cellular ROS generation, restoring enzymatic and non-enzymatic antioxidants, as well as improving liver function enzymes. Also, GA can ameliorate the abnormality of biochemical parameters and histopathological changes in DIC-induced liver toxicity.
